# Clinical characteristics and short-term outcomes of patients with critical acute pulmonary embolism requiring extracorporeal membrane oxygenation: from the COMMAND VTE Registry-2

**DOI:** 10.1186/s40560-024-00755-x

**Published:** 2024-11-05

**Authors:** Kensuke Takabayashi, Yugo Yamashita, Takeshi Morimoto, Ryuki Chatani, Kazuhisa Kaneda, Yuji Nishimoto, Nobutaka Ikeda, Yohei Kobayashi, Satoshi Ikeda, Kitae Kim, Moriaki Inoko, Toru Takase, Shuhei Tsuji, Maki Oi, Takuma Takada, Kazunori Otsui, Jiro Sakamoto, Yoshito Ogihara, Takeshi Inoue, Shunsuke Usami, Po-Min Chen, Kiyonori Togi, Norimichi Koitabashi, Seiichi Hiramori, Kosuke Doi, Hiroshi Mabuchi, Yoshiaki Tsuyuki, Koichiro Murata, Hisato Nakai, Daisuke Sueta, Wataru Shioyama, Tomohiro Dohke, Ryusuke Nishikawa, Koh Ono, Takeshi Kimura, On behalf of the COMMAND VTE Registry-2 Investigators

**Affiliations:** 1 Department of Cardiology, Hirakata Kohsai Hospital, 1-2-1, Fujisakashigashimachi, Hirakata-shi, Osaka, 573-0153 Japan; 2 Department of Cardiovascular Medicine, Graduate School of Medicine, Kyoto University, Kyoto, Japan; 3 Department of Clinical Epidemiology, Hyogo College of Medicine, Nishinomiya, Japan; 4 Department of Cardiovascular Medicine, Kurashiki Central Hospital, Kurashiki, Japan; 5 Department of Cardiology, Hyogo Prefectural Amagasaki General Medical Center, Amagasaki, Japan; 6 Division of Cardiovascular Medicine, Toho University Ohashi Medical Center, Tokyo, Japan; 7 Department of Cardiovascular Center, Osaka Red Cross Hospital, Osaka, Japan; 8 Department of Cardiovascular Medicine, Nagasaki University Graduate School of Biomedical Sciences, Nagasaki, Japan; 9 Department of Cardiovascular Medicine, Kobe City Medical Center General Hospital, Kobe, Japan; 10 Cardiovascular Center, The Tazuke Kofukai Medical Research Institute, Kitano Hospital, Osaka, Japan; 11 Department of Cardiology, Kinki University Hospital, Osaka, Japan; 12 Department of Cardiology, Japanese Red Cross Wakayama Medical Center, Wakayama, Japan; 13 Department of Cardiology, Japanese Red Cross Otsu Hospital, Otsu, Japan; 14 Department of Cardiology, Tokyo Women’s Medical University, Tokyo, Japan; 15 Department of General Internal Medicine, Kobe University Hospital, Kobe, Japan; 16 Department of Cardiology, Tenri Hospital, Tenri, Japan; 17 Department of Cardiology and Nephrology, Mie University Graduate School of Medicine, Tsu, Japan; 18 Department of Cardiology, Shiga General Hospital, Moriyama, Japan; 19 Department of Cardiology, Kansai Electric Power Hospital, Osaka, Japan; 20 Department of Cardiology, Osaka Saiseikai Noe Hospital, Osaka, Japan; 21 Division of Cardiology, Faculty of Medicine, Nara Hospital, Kinki University, Ikoma, Japan; 22 Department of Cardiovascular Medicine, Gunma University Graduate School of Medicine, Maebashi, Japan; 23 Department of Cardiology, Kokura Memorial Hospital, Kokura, Japan; 24 Department of Cardiology, National Hospital Organization Kyoto Medical Center, Kyoto, Japan; 25 Department of Cardiology, Koto Memorial Hospital, Higashiomi, Japan; 26 Division of Cardiology, Shimada General Medical Center, Shimada, Japan; 27 Department of Cardiology, Shizuoka City Shizuoka Hospital, Shizuoka, Japan; 28 Department of Cardiovascular Medicine, Sugita Genpaku Memorial Obama Municipal Hospital, Obama, Japan; 29 Department of Cardiovascular Medicine, Graduate School of Medical Sciences, Kumamoto University, Kumamoto, Japan; 30 Department of Cardiovascular Medicine, Shiga University of Medical Science, Otsu, Japan; 31 Division of Cardiology, Kohka Public Hospital, Koka, Japan

**Keywords:** ECMO, Acute pulmonary embolism, Surgical pulmonary embolectomy, Prognosis, Mortality, Major bleeding

## Abstract

**Background:**

Extracorporeal membrane oxygenation (ECMO) might be required as a treatment option in patients with critical pulmonary embolism (PE). However, the clinical features and outcomes of the use of ECMO for critical acute PE are still limited. The present study aimed to clarify the clinical characteristics, management strategies and outcomes of patients with acute PE requiring ECMO in the current era using data from a large-scale observational database.

**Methods:**

We analyzed the data of the COMMAND VTE Registry-2: a physician-initiated, multicenter, retrospective cohort study enrolling consecutive patients with acute symptomatic venous thromboembolism (VTE). Among 2035 patients with acute symptomatic PE, there were 76 patients (3.7%) requiring ECMO.

**Results:**

Overall, the mean age was 58.4 years, and 34 patients (44.7%) were men. Cardiac arrest or circulatory collapse at diagnosis was reported in 67 patients (88.2%). The 30-day incidence of all-cause death was 30.3%, which were all PE-related deaths. The 30-day incidence of major bleeding was 54.0%, and the vast majority of bleedings were procedure site-related bleeding events and surgery-related bleeding (22.4%). The 30-day incidence of all-cause death was 6.3% in 16 patients with surgical intervention, 43.8% in 16 patients with catheter intervention, 25.0% in 16 patients with thrombolytic therapy, and 39.3% in 28 patients with anticoagulation only.

**Conclusions:**

The current large real-world VTE registry in Japan revealed clinical features and outcomes of critical acute PE requiring ECMO in the current era, which suggested several unmet needs for future clinical trials.

**Supplementary Information:**

The online version contains supplementary material available at 10.1186/s40560-024-00755-x.

## Background

Pulmonary embolism (PE) is a serious clinical presentation of venous thromboembolism (VTE). Extracorporeal membrane oxygenation (ECMO) could be a treatment option for critical acute PE when cardiac arrest or circulatory collapse is impending or when hemodynamic instability continues despite other treatments [1, 2]. Actually, ECMO has been considered as a bridge therapy to intensive treatment for clot resolution including surgical or catheter embolectomy and systemic thrombolysis [3–5]. A previous study reported that ECMO therapy was associated with a lower mortality risk in critical PE patients and there was a trend toward increasing use of ECMO for patients with massive PE [6], although mortality risk for patients receiving ECMO was still high [6–8]. However, because use of ECMO for critical acute PE has not been so common in daily clinical practice, there is a scarcity of data on patients with PE who were treated with ECMO, which could be important in understanding the unsolved issues and unmet needs in the current treatment of massive PE. Therefore, the present study aimed to clarify the clinical characteristics, management strategies and outcomes in patients with critical acute PE requiring ECMO in a large-scale observational database in Japan.

## Methods

### Study design

The COMMAND VTE Registry-2 is a physician-initiated, multicenter, retrospective cohort study enrolling consecutive patients with acute symptomatic VTE objectively confirmed by imaging examination or by autopsy among 31 participating centers in Japan between January, 2015 and August, 2020 after the introduction of direct oral anticoagulants (DOACs) for VTE in Japan. The registry design was previously reported in detail [9]. Just briefly, we registered consecutive patients who met the definitions of acute symptomatic VTE diagnosed within 31 days after symptoms onset during the study period [10]. The study was conducted in accordance with the principles of the Declaration of Helsinki. The relevant review boards or ethics committee in all participating centers approved the research protocol (Supplementary Appendix 1, Supplementary Appendix Table). We obtained informed consent in the form of an opt-out on the website of each hospital, because we only used clinical information obtained during routine clinical practice. This method was concordant with the guidelines for epidemiological studies issued by the Ministry of Health, Labor, and Welfare in Japan.

### Study population

**Fig. 1:**
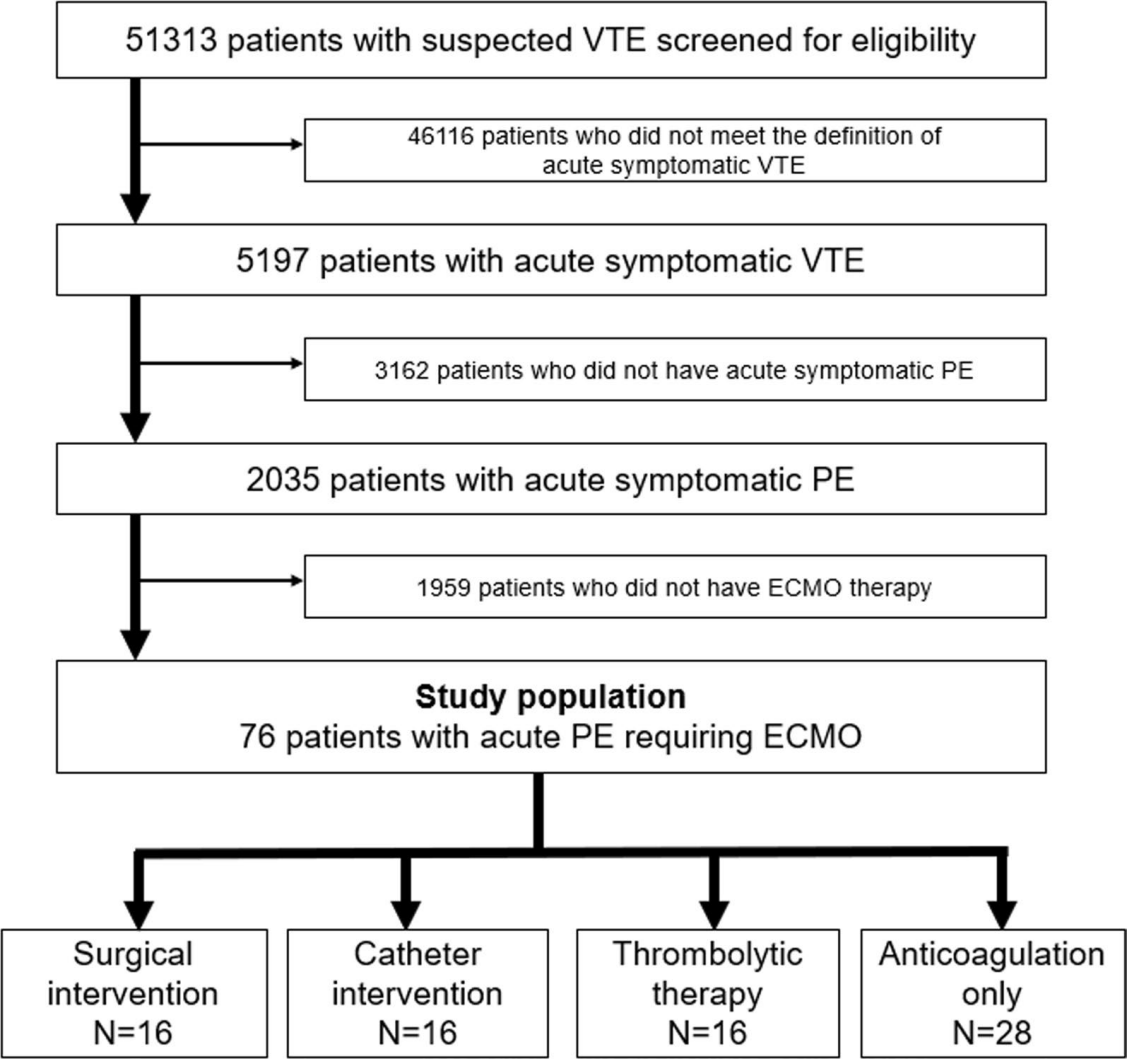
Patients flowchart in this study. *ECMO* extracorporeal membrane oxygenation, *PE* pulmonary embolism, *VTE* venous thromboembolism

After screening 51,313 patients with suspected VTE for eligibility through chart review by the physicians in each participating center, a total of 5197 patients with acute symptomatic VTE were enrolled in the registry. After excluding 3162 patients without acute symptomatic PE and 1959 PE patients without ECMO use, the current study population consisted of 76 (3.7%) patients with acute PE requiring ECMO (Fig. 1). To investigate the difference depending on different treatment strategies, we divided the current study population into the following 4 subgroups; surgical pulmonary embolectomy (surgical intervention), catheter pulmonary embolectomy (catheter intervention), systemic thrombolysis (thrombolytic therapy) and anticoagulation only. Anticoagulation therapy was defined as oral or parenteral anticoagulation therapy (warfarin, DOAC, or heparin). When multiple treatments were conducted, we exclusively classified them in the following order based on the degree of invasiveness; surgical intervention > catheter intervention > thrombolytic therapy > anticoagulation only. Furthermore, to investigate the difference in baseline characteristics and treatment depending on the vital status at 30 days, we divided the current study population into the survivor and non-survivor groups at 30 days.

### Data collection and definitions of patient characteristics

Data on patient characteristics were collected from the hospital charts or hospital databases according to the prespecified definitions using an electronic case report form in a web-based database system. The physicians in each center were responsible for the entry of data, which were automatically checked for missing or contradictory input and values out of the expected range. The general office of the registry performed additional editing checks.

Patients with active cancer were defined at the time of VTE diagnosis as those receiving treatment for cancer such as chemotherapy or radiotherapy, those scheduled to undergo cancer surgery, those with metastasis to other organs, or those with terminal cancer defined as an expected life expectancy of 6 months or less. History of major bleeding was diagnosed if the patient had a history of International Society of Thrombosis and Hemostasis (ISTH) major bleeding, which consisted of fatal bleeding, symptomatic bleeding in a critical area or organ, and bleeding causing a reduction in the hemoglobin level by at least 2 g/dL or leading to transfusion of at least 2 units of whole blood or red cells. ECMO use at hospital arrival initiated immediately at diagnosis after hospital arrival. In the other group, ECMO was used as the condition worsened after starting initial treatment without ECMO. The detailed definitions of other patient characteristics are provided in Supplementary Appendix 2.

### Clinical outcomes

The primary outcome was all-cause death during the 30-day period. The secondly outcome was major bleeding. PE-related death (fatal PE) was adjudicated if it was confirmed by autopsy or if death followed a clinically severe PE, either initially or after recurrent PE events. Major bleeding was defined as ISTH major bleeding [11]. The members of the independent clinical event committee, who were blinded to the patient characteristics, reviewed the detailed clinical courses, and adjudicated the clinical events (Supplementary Appendix 3). If there was inconsistency, final adjudication for clinical events was made based on the full consensus of the committee [9].

### Statistical analysis

Considering the small number of patients with ECMO use, we focused on the descriptive statistics in this manuscript. Categorical variables were described as numbers and percentages (%), and continuous variables were described as means and standard deviation or medians and interquartile range (IQR) based on their distributions. The Kaplan–Meier method was used to evaluate the cumulative incidences of clinical outcomes, and we assessed the differences by the log-rank test. Exploratory, we performed univariate logistic regression analysis to evaluate the potential influence of several variables on all-cause death at 30 days, and calculated the odds ratios (OR) with 95% confidence intervals (CI).

All statistical analyses were performed using JMP version 14 (SAS Institute, Cary, NC, USA).

## Results

### Clinical characteristics

Overall, the mean age was 58.4 years, 34 (44.7%) patients were men, and the mean body mass index was 24.3 kg/m^2^ (Table 1). Four patients (5.6%) had history of VTE and 5 patients (6.6%) had history of major bleeding. Among 76 patients with ECMO, 16 (21.0%) patients were treated with surgical intervention. Patients who developed PE out of hospital accounted for 60 (79.0%) (Table 2). Patients who presented with cardiac arrest or collapse at diagnosis accounted for 67 (88.2%), and patients who received ECMO at hospital arrival accounted for 61 (80.3%). The median D-dimer level at diagnosis was 28.1 µg/mL and the median BNP level was 225.6 pg/mL.

**Table 1:** Clinical characteristics

	Overall	Surgical intervention	Catheter intervention	Thrombolytic therapy	Anticoagulation only
Number	76	16	16	16	28
Baseline characteristics					
Gender, male	34 (44.7%)	5 (31.3%)	7 (43.8%)	9 (56.3%)	13 (46.4%)
Age (years)	58.4 ± 14.3	61.7 ± 11.8	55.5 ± 14.8	59.6 ± 17.1	57.6 ± 14.0
BMI (kg/m^2^)	24.3 ± 4.5	24.7 ± 4.5	23.8 ± 4.2	23.8 ± 4.0	24.7 ± 5.0
BMI ≥ 30 kg/m^2^	7 (9.2%)	2 (12.5%)	0 (0.0%)	0 (0.0%)	5 (17.9%)
Comorbidity					
History of VTE	4 (5.3%)	0 (0.0%)	1 (6.3%)	2 (12.5%)	1 (3.6%)
History of PE	2 (2.6%)	0 (0.0%)	0 (0.0%)	1 (6.3%)	1 (3.6%)
History of DVT	4 (5.3%)	0 (0.0%)	1 (6.3%)	2 (12.5%)	1 (3.6%)
Chronic heart disease	11 (14.5%)	2 (12.5%)	5 (31.3%)	2 (12.5%)	2 (7.1%)
History of stroke	2 (2.6%)	0 (0.0%)	1 (6.3%)	1 (6.3%)	0 (0.0%)
COPD/asthma	4 (5.3%)	0 (0.0%)	2 (12.5%)	0 (0.0%)	2 (7.1%)
History of major bleeding	5 (6.6%)	2 (12.5%)	1 (6.3%)	1 (6.3%)	1 (3.6%)
Hypertension	32 (42.1%)	4 (25.0%)	7 (43.8%)	9 (56.3%)	12 (42.9%)
Diabetes	11 (14.5%)	0 (0.0%)	3 (18.8%)	3 (18.8%)	5 (17.9%)
Dyslipidemia	13 (17.1%)	2 (12.5%)	3 (18.8%)	3 (18.8%)	5 (17.9%)
Chronic kidney disease	14 (18.4%)	2 (12.5%)	3 (18.8%)	4 (25.0%)	5 (17.9%)
Dialysis	1 (1.3%)	0 (0.0%)	0 (0.0%)	0 (0.0%)	1 (3.6%)
Autoimmune disease	2 (2.6%)	0 (0.0%)	1 (6.3%)	0 (0.0%)	1 (3.6%)
Congenital coagulation defects	7 (9.2%)	0 (0.0%)	1 (6.3%)	3 (18.8%)	3 (10.7%)
Active cancer	6 (7.9%)	2 (12.5%)	1 (6.3%)	0 (0.0%)	3 (10.7%)

*BMI* body mass index, *VTE* venous thromboembolism, *PE* pulmonary embolism, *DVT* deep vein thrombosis, *COPD* chronic obstructive pulmonary disease. The detailed definition of comorbidities is shown in supplementary appendix 2

**Table 2:** Initial presentation and laboratory data at diagnosis

	Overall	Surgical intervention	Catheter intervention	Thrombolytic therapy	Anticoagulation only
Number	76	16	16	16	28
Initial presentation					
Out-of-hospital onset	60 (79.0%)	11 (68.8%)	12 (75.0%)	15 (93.8%)	22 (78.6%)
Arrest/collapse at diagnosis	67 (88.2%)	12 (75.0%)	13 (81.3%)	15 (93.8%)	27 (96.4%)
Arrest at diagnosis	52 (68.4%)	10 (62.5%)	11 (68.8%)	7 (43.8%)	24 (85.7%)
Collapse at diagnosis	15 (19.7%)	2 (12.5%)	2 (12.5%)	8 (50.0%)	3 (10.7%)
Concomitant DVT	41 (54.0%)	12 (75.0%)	10 (62.5%)	9 (56.3%)	10 (35.7%)
ECMO use at hospital arrival	61 (80.3%)	13 (81.3%)	12 (75.0%)	12 (75.0%)	24 (85.7%)
Laboratory data at diagnosis					
WBC (/μL)	11,250 [9425–15,000]	10,150 [8937–19,450]	12,050 [8550–15,750]	11,350 [9900–14,100]	11,250 [9425–15,067]
Hemoglobin (g/dL)	12.0 [10.1–14.3]	11.0 [8.9–13.3]	13.3 [10.0–14.6]	14.2 [11.8–16.0]	11.6 [9.7–13.7]
Platelet (10^9^/μL)	16.7 [11.6–23.7]	17.0 [10.5–24.3]	19.2 [14.6–22.6]	16.1 [12.4–22.3]	15.8 [10.5–25.6]
Total bilirubin (mg/dL)	0.6 [0.4–0.8]	0.8 [0.7–1.45]	0.6 [0.4–0.8]	0.6 [0.5–0.8]	0.6 [0.3–0.8]
AST (IU/L)	111 [43–230]	77 [41–144]	142 [35–252]	120 [29–222]	122 [59–274]
ALT (IU/L)	80 [39–177]	47 [20–113]	85 [67–182]	54 [24–162]	114 [52–233]
Serum creatinine (mg/dL)	1.03 [0.83–1.33]	0.94 [0.78–1.10]	0.96 [0.79–1.16]	1.13 [0.93–1.33]	1.09 [0.86–1.45]
eGFR (mL/min/1.73m^2^)	49.9 [40.1–60.3]	50.1 [43.6–60.8]	51.0 [38.6–70.5]	52.9 [40.7–56.4]	43.5 [38.7–58.6]
D-dimer (μg/mL)	28.1 [13–49]	33.2 [12.8–57.3]	26.3 [14.3–33.5]	21.5 [10.6–66.0]	34.5 [9.4–51.9]
BNP (pg/mL)	225.6 [70.1–592.7]	605.2 [58.6–892.8]	330.1 [32.5–918.5]	307.7 [29.5–713.3]	183.9 [83.0–460.5]

*DVT* deep vein thrombosis, *ECMO* extracorporeal membrane oxygenation, *WBC* white blood cell, *AST* aspartate aminotransferase, *ALT* alanine aminotransferase, *eGFR* estimated glomerular filtration rate, *BNP* brain natriuretic peptide. ECMO use at hospital arrival initiated immediately at diagnosis after hospital arrival. In the other group, ECMO was used as the condition worsened after starting initial treatment without ECMO

### Clinical outcomes at 30 days

**Fig. 2:**
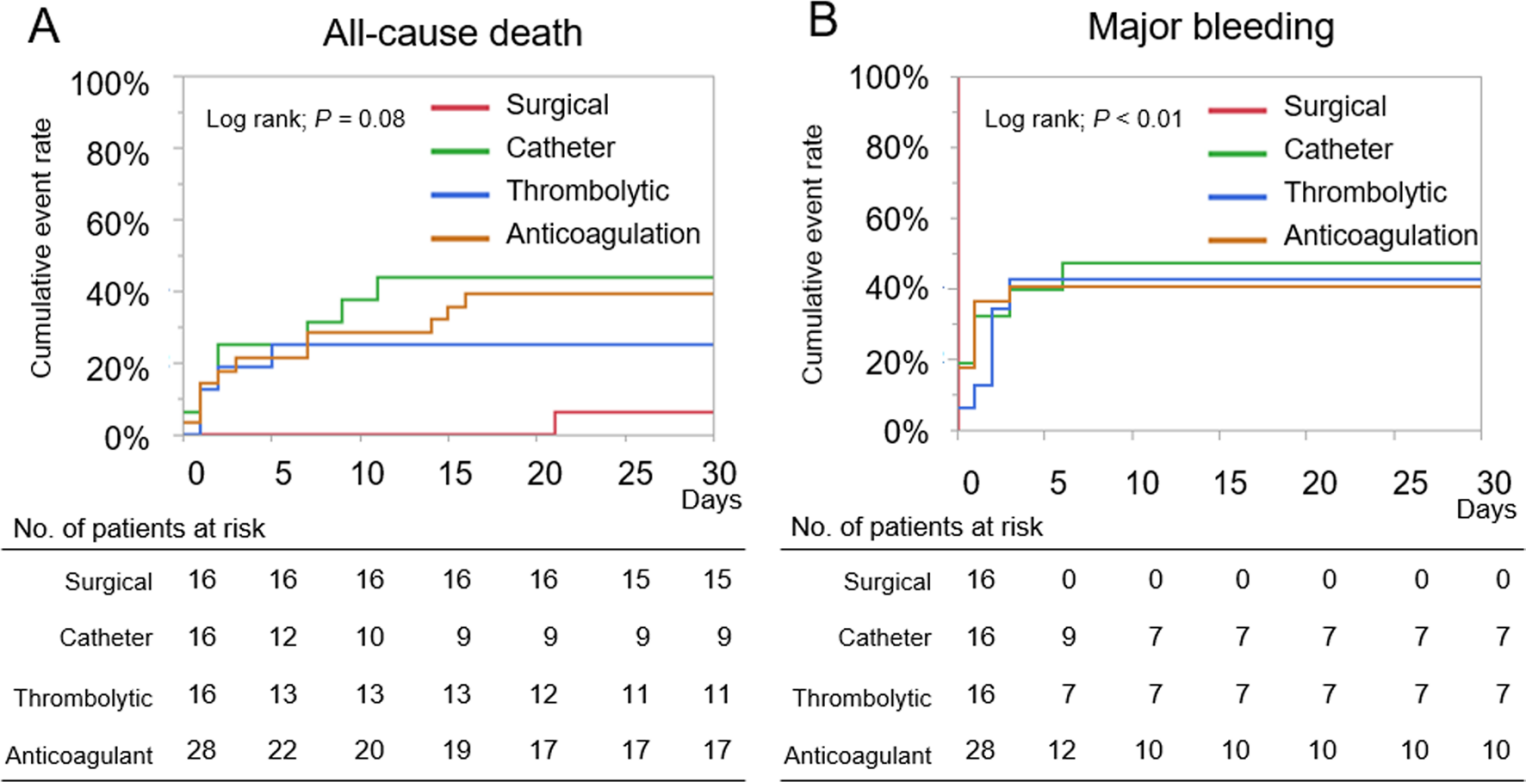
The Kaplan–Meier curves for **A** all-cause death and **B** major bleeding according to the treatment strategies

Overall, 17 (30.3%) patients died at 30 days, and all of the deaths were due to PE (PE-related death) (Table 3). Major bleeding occurred in 41 (54.0%) at 30 days, and the most common bleeding site was procedure site bleeding and surgery-related bleeding (*N* = 17). All patients who received surgical intervention had surgery-related major bleeding events leading to transfusion of 2 or more units of red blood cells during the surgical procedures or within 48 h after surgery, however the surgical intervention group had a numerically lower 30-day incidence of all-cause death compared with other groups (surgical intervention: 6.3%, catheter intervention: 43.8%, thrombolytic therapy: 25.0%, and anticoagulation only: 39.3%). The Kaplan–Meier curves for all-cause death and major bleeding according to the treatment strategies are described in Fig. 2. Most of all-cause death and major bleeding events occurred within 10 and 5 days, respectively.

**Table 3:** Clinical outcomes at 30 days

	Overall	Surgical intervention	Catheter intervention	Thrombolytic therapy	Anticoagulation only
Number	76	16	16	16	28
All-cause death	23 (30.3%)	1 (6.3%)	7 (43.8%)	4 (25.0%)	11 (39.3%)
PE-related death	23 (30.3%)	1 (6.3%)	7 (43.8%)	4 (25.0%)	11 (39.3%)
Major bleeding	41 (54.0%)	16 (100%)*	7 (43.8%)	6 (37.5%)	12 (42.9%)
Intracranial bleeding	3 (3.9%)	0 (0.0%)	0 (0.0%)	1 (6.3%)	2 (7.1%)
Respiratory bleeding	2 (2.6%)	0 (0.0%)	1 (6.3%)	1 (6.3%)	0 (0.0%)
Thoracic cavity/abdominal cavity	2 (2.6%)	0 (0.0%)	0 (0.0%)	1 (6.3%)	1 (3.6%)
Procedure site bleeding	17 (22.4%)	0 (0.0%)	6 (37.5%)	3 (18.8%)	8 (28.6%)
Surgery-related bleeding	17 (22.4%)	16 (100%)	0 (0.0%)	0 (0.0%)	1 (3.6%)

*PE* pulmonary embolism. The detailed definition of clinical outcomes is shown in supplementary appendix 4

*All major bleeding events were bleeding events leading to transfusion of 2 or more units of red blood cells during the surgical procedures or within 48 h after surgery

### Clinical features comparing the survivors and non-survivors at 30 days

The baseline characteristics, comorbidity, initial presentation, and laboratory data were generally similar between the survivor and non-survivor groups except for brain natriuretic peptide, which was much lower in the survivor group than in the non-survivor group (Table 4). As for treatment, the survivor group included a much higher proportion of surgical intervention than the non-survivor group (28.3 vs. 4.4%, *P* = 0.009). Univariate regression analysis demonstrated that surgical intervention was significantly associated with a lower risk of 30-day all-cause death (OR, 0.12; 95% CI, 0.01–0.93).

**Table 4:** Clinical characteristics comparing the survivor and non-survivor at 30 days

	Survivor	Non-survivor	Univariate odds ratio (95% CI)
Number	53	23	
Baseline characteristics			
Gender, male	23 (43.4%)	11 (47.8%)	1.20 (0.44–3.19)
Age (years)	59.3 ± 13.6	56.4 ± 16.0	0.99 (0.95–1.02)
BMI (kg/m^2^)	24.0 ± 4.0	25.1 ± 5.5	1.06 (0.94–1.18)
BMI ≥ 30 kg/m^2^	3 (5.7%)	4 (17.4%)	3.51 (0.72–17.16)
Comorbidity			
History of VTE	3 (5.7%)	1 (4.4%)	0.76 (0.07–7.69)
History of PE	1 (1.9%)	1 (4.4%)	2.36 (0.14–39.51)
History of DVT	3 (5.7%)	1 (4.4%)	0.76 (0.07–7.69)
Chronic heart disease	5 (9.4%)	6 (26.1%)	3.39 (0.91–12.55)
History of stroke	2 (3.8%)	0 (0%)	N/A
COPD/asthma	1 (1.9%)	3 (13.0%)	7.8 (0.77–79.56)
History of major bleeding	3 (5.7%)	2 (8.7%)	1.59 (0.25–10.20)
Hypertension	23 (43.4%)	9 (39.1%)	0.84 (0.31–2.28)
Diabetes	8 (15.1%)	3 (13.0%)	0.84 (0.20–3.52)
Dyslipidemia	11 (20.8%)	2 (8.7%)	0.36 (0.07–1.79)
Chronic kidney disease	9 (17.0%)	5 (21.7%)	1.36 (0.40–4.61)
Dialysis	1 (1.9%)	0 (0%)	N/A
Autoimmune disease	0 (0%)	2 (8.7%)	N/A
Congenital coagulation defects	4 (7.6%)	3 (13.0%)	1.84 (0.38–8.96)
Active cancer	3 (5.7%)	3 (13.0%)	2.50 (0.46–13.44)
Initial presentation			
Out-of-hospital onset	40 (75.5%)	20 (87.0%)	2.17 (0.55–8.49)
Arrest/collapse at diagnosis	48 (90.6%)	19 (82.6%)	0.49 (0.12–2.04)
Arrest at diagnosis	35 (66.0%)	17 (73.9%)	1.46 (0.49–4.34)
Collapse at diagnosis	13 (24.5%)	2 (8.7%)	0.29 (0.06–1.42)
ECMO use at hospital arrival	44 (83.0%)	17 (73.9%)	0.57 (0.18–1.88)
Laboratory data			
WBC (/μL)	13,200 [1000–15190]	10,800 [9125–14750]	1.00 (0.99–1.00)
Hemoglobin (g/dL)	12 [9.7–14.3]	12.1 [10.9–14.6]	1.04 (0.88–1.23)
Platelet (10^9^/μL)	16.2 [12.6–24.4]	17.3 [9.3–20.4]	0.98 (0.93–1.04)
Serum creatinine (mg/dL)	1.01 [0.81–1.19]	1.09 [0.92–1.38]	1.18 (0.70–1.98)
eGFR (mL/min/1.73m^2^)	50.5 [40.8–60.7]	48.1 [36.3–56.6]	0.98 (0.95–1.01)
Total bilirubin (mg/dL)	0.6 [0.4–0.9]	0.65 [0.4–0.8]	1.03 (0.69–1.54)
D-dimer (μg/mL)	28.8 [12.5–53.9]	23.4 [12.8–36.8]	0.99 (0.98–1.01)
BNP (pg/mL)	143.8 [36.5–4442.6]	800.1 [230.9–1365.1]	1.00 (1.00–1.01)
Treatment			
Surgical intervention	15 (28.3%)	1 (4.4%)	0.12 (0.01–0.93)
Catheter intervention	10 (18.9%)	7 (30.4%)	1.88 (0.61–5.79)
Thrombolytic therapy	13 (24.5%)	8 (34.8%)	1.64 (0.57–4.75)
Anticoagulation only	17 (32.1%)	11 (47.8%)	1.94 (0.71–5.28)

*BMI* body mass index, *VTE* venous thromboembolism, *PE* pulmonary embolism, *DVT* deep vein thrombosis, *COPD* chronic obstructive pulmonary disease, *ECMO* extracorporeal membrane oxygenation, *WBC* white blood cell, *eGFR* estimated glomerular filtration rate, *BNP* brain natriuretic peptide, *N/A* not applicable. The detailed definition of comorbidities is shown in supplementary appendix 2. The odds ratio indicates the risk of death based on the number of survivors

## Discussion

The present study demonstrated the detailed epidemiological snapshot of patients with critical acute PE requiring ECMO in Japan. The present study from Japan showed a relatively higher prevalence of ECMO for critical PE (3.7%) compared with reports from the Western countries; previous studies reported that prevalence of ECMO was 0.28% among high-risk PE in the United States, and 0.2% among acute PE in Germany [6, 7]. The present study also showed that patients with ECMO were older (mean age: 58.4 years) and less often men (44.7%) than those in the Western countries; previous studies reported that the mean age was 42 years and the proportion of men was 67.6% in the United States, and the median age was 55 years and the proportion of men was 61.8% in Germany [6, 7]. Other recent reports between 2015 and 2022 in the United States showed that about 2.2% of PE patients with high risk received ECMO therapy [12, 13]. Another study showed that patients with ECMO for acute PE had better outcomes compared with those supported for other indications [14]. There was no significant differences of mortality and bleeding events rate between patients treated with ECMO after systemic thrombosis and those who were not [15]. The use of ECMO on high-risk patients with pulmonary embolism may have become more common in recent years.

The proportion of surgical intervention among patients with ECMO in the present study (21.1%) was comparable to that in United States (17.1%) and in Germany (20.4%) [6, 7]. Notably, the present study also showed a lower incidence rate of mortality (30.3%) in patients with critical acute PE requiring ECOM compared with that in United States (61.6%) and in Germany (61.8%) [6, 7]. In our study, 85.7% of patients had arrest or collapse when ECMO was started. In German cohort, 55.5% of patients had shock and 45.2% of patients needed cardiopulmonary resuscitation [7]. A recent study in France showed 79% of high-risk PE patients with ECMO had arrest at ECMO use and 90-day mortality rate was 59% [15]. Another study showed hospital mortality rate of 47.2% in PE patients with ECMO between 2010 and 2019 [14]. Mortality rate of patients requiring ECMO for acute PE remains still high. These differences could be partly due to the difference in demographics, practice pattern, emergency medical system, and access to medical care.

The current Japanese Circulation Society (JCS) guidelines have recommended to consider ECMO use for patients with cardiac arrest or collapse due to critical PE, and combination with anticoagulation and thrombolysis therapy, or surgical embolectomy, or catheter-directed treatment depending on the available treatment strategies in each institution [16]. A previous study reported that additional ECMO use for patients with failed fibrinolysis resulting in no reperfusion was associated with unfavorable outcomes compared with ECMO use and surgical intervention [8]. Another study also reported that ECMO was a valuable supportive treatment in conjunction with reperfusion treatment, but not as a stand-alone treatment especially for patients with arrest [13]. Present study showed patients with catheter intervention, thrombolytic therapy and anticoagulation only were at a numerically higher risk of mortality compared with surgical intervention, which seemed to be consistent with the previous studies [7, 17]. These findings might suggest that these treatment options other than surgical intervention seemed not to be enough for cases of huge clots resulting in cardiac arrest or collapse. When the amount of thrombus is extremely large, there is concern that sufficient effects cannot be expected from thrombolytic therapy or anticoagulation only and that there is an increased risk of bleeding complications. Surgical intervention under ECMO might be the most effective treatment option in terms of removal of clots. Actually, a previous study reported the utility of a protocolized strategy for acute PE including appropriate surgical intervention for massive PE [18]. On the other hand, availability of surgical intervention could vary widely depending on each institution. Additional treatments for patients with ECMO depended on the patients’ background, medical institution system, and predicted neurological prognosis. In our study, patients with anticoagulation only had the highest rate of arrest at diagnosis (85.7%) in each treatment. In Japan, there are no approved specific devices for use in pulmonary artery thrombus aspiration or thrombus disruption. Thus, alternative treatment options with more easy availability could be unmet needs for critical PE requiring ECMO, which might include upcoming new catheter treatment [19].

The present study had several limitations. First, the study was based on an observational study and treatment strategies, such as implementation of ECMO and several invasive treatments, were determined at the discretion of the attending physicians. Indications for ECMO have been strongly influenced by economics and other non-medical factors. Thus, the results were hypothesis generating and should be interpreted with caution. Second, the absolute number of patients with ECMO was small (*N* = 76), although it was derived from a large observational database of patients with VTE. Due to lack of adequate statistical power, we could not conduct detailed analyses including multivariable analysis. Third, there is no detailed data of the amount of thrombus in pulmonary artery, its distribution or neurological outcomes. Most of the patients with ECMO had arrest or collapse in this study. Our study, like others, included a large number of patients with severe acute pulmonary embolism. Therefore, the results should be regarded as exploratory.

## Conclusions

The current large real-world VTE registry in Japan revealed clinical features and outcomes of critical acute PE requiring ECMO in the current era, which suggested several unmet needs for future clinical trials.

## Supplementary Information

Additional file 1.

Additional file 2.

## Data Availability

The data, analytic methods, and study materials will not be made available to other researchers for purposes of reproducing the results or replicating the procedure. However, if the relevant review board or ethics committee approve data sharing and all investigators of the COMMAND VTE Registry-2 provide consent, the deidentified participant data will be shared on a request basis through the principal investigator. Study protocol will also be available. The data will be shared as Excel files via e-mail during the proposed investigation period.

## Abbreviations

PE: Pulmonary embolism
VTE: Venous thrombo-embolism
ECMO: Extracorporeal membrane oxygenation
DOACs: Direct oral anticoagulants
ISTH: International Society of Thrombosis and Hemostasis
IQR: Interquartile range
OR: Odds ratios
CI: Confidence intervals
