# Supplementary material for: Clinical characteristics and short-term outcomes of patients with critical acute pulmonary embolism requiring extracorporeal membrane oxygenation: from the COMMAND VTE Registry-2
Source: J Intensive Care. 2024 Nov 5;12:45. doi: 10.1186/s40560-024-00755-x (PMC11536536; doi:10.1186/s40560-024-00755-x)
Supplement: Supplementary file 1 — Additional file 1. [file 40560_2024_755_MOESM1_ESM.docx]

**Supplementary Materials**

**Supplementary Appendix 1: List of participating centers and investigators**

- Department of Cardiovascular Medicine, Graduate School of Medicine, Kyoto University, Kyoto, Japan: Kazuhisa Kaneda, Ryusuke Nishikawa, Yugo Yamashita (Kyoto University Hospital Ethics Committee; the approval number R3082)
- Department of Cardiovascular Medicine, Kurashiki Central Hospital, Kurashiki, Japan: Ryuki Chatani, Kazunori Mushiake, Kazushige Kadota (Kurashiki Central Hospital Ethics Committee; the approval number 3684)
- Department of Cardiology, Hyogo Prefectural Amagasaki General Medical Center, Amagasaki, Japan: Yuji Nishimoto, Yukihito Sato (Hyogo Prefectural Amagasaki General Medical Center Ethics Committee; the approval number 2021-3-50)
- Division of Cardiovascular Medicine, Toho University Ohashi Medical Center, Tokyo, Japan: Nobutaka Ikeda, Katsushi Amemiya, Masato Nakamura (Toho University Ohashi Medical Center Ethics Committee; the approval number H21053)
- Department of Cardiovascular Center, Osaka Red Cross Hospital, Osaka, Japan: Yohei Kobayashi, Ren Kimura, Tsukasa Inada (Osaka Red Cross Hospital Ethics Committee; the approval number J-0268)
- Department of Cardiovascular Medicine, Nagasaki University Graduate School of Biomedical Sciences, Nagasaki, Japan: Satoshi Ikeda, Yuki Ueno, Koji Maemura (Nagasaki University Ethics Committee; the approval number 21091305)
- Department of Cardiovascular Medicine, Kobe City Medical Center General Hospital, Kobe, Japan: Kitae Kim, Ryo Shigeno, Yutaka Furukawa (Kobe City Medical Center General Hospital Ethics Committee; the approval number 2021-09-17)
- Cardiovascular Center, The Tazuke Kofukai Medical Research Institute, Kitano Hospital, Osaka, Japan: Moriaki inoko, Shinya Ito (Kitano Hospital Ethics Committee; the approval number P211000100)
- Department of Cardiology, Kinki University Hospital, Osaka, Japan: Toru Takase, Gaku Nakazawa (Kinki University Ethics Committee; the approval number R03-170)
- Department of Cardiology, Japanese Red Cross Wakayama Medical Center, Wakayama, Japan: Shuhei Tsuji, Mamoru Toyofuku (Japanese Red Cross Wakayama Medical Center Ethics Committee; the approval number 2021-8-18)
- Department of Cardiology, Japanese Red Cross Otsu Hospital, Otsu, Japan: Maki Oi, Kazuaki Kaitani (Japanese Red Cross Otsu Hospital Ethics Committee; the approval number 650)
- Department of Cardiology, Tokyo Women's Medical University, Tokyo, Japan: Takuma Takada, Kentaro Jujo, Nobuhisa Hagiwara (Tokyo Women's Medical University Ethics Committee; the approval number 2021-0101)
- Department of General Internal Medicine, Kobe University Hospital, Kobe, Japan: Kazunori Otsui, Kenta Mori (Kobe University Hospital Ethics Committee; the approval number B210264)
- Department of Cardiology, Tenri Hospital, Tenri, Japan: Jiro Sakamoto, Toshihiro Tamura (Tenri Hospital Ethics Committee; the approval number 1228)
- Department of Cardiology and Nephrology, Mie University Graduate School of Medicine, Tsu, Japan: Yoshito Ogihara, Toru Sato, Kaoru Dohi (Mie University Hospital Ethics Committee; the approval number H2021-192)
- Department of Cardiology, Shiga General Hospital, Moriyama, Japan: Takeshi Inoue, Tetsuya Nadahama, Kunihiko Kosuga (Shiga General Hospital Ethics Committee; the approval number 20211119-02)
- Department of Cardiology, Kansai Electric Power Hospital, Osaka, Japan: Shunsuke Usami, Katsuhisa Ishii (Kansai Electric Power Hospital Ethics Committee; the approval number 21-067)
- Department of Cardiology, Osaka Saiseikai Noe Hospital, Osaka, Japan: Po-Min Chen, Toshiaki Izumi (Osaka Saiseikai Noe Hospital Ethics Committee; the approval number 20211103)
- Division of Cardiology, Nara Hospital, Kinki University Faculty of Medicine, Ikoma, Japan: Kiyonori Togi, Manabu Shirotani (Nara Hospital, Kinki University Faculty of Medicine Ethics Committee; the approval number 652)
- Department of Cardiology, Mitsubishi Kyoto Hospital, Kyoto, Japan: Kazuhisa Kaneda, Takafumi Yokomatsu (Mitsubishi Kyoto Hospital Ethics Committee; the approval number 21-28)
- Department of Cardiovascular Medicine, Gunma University Graduate School of Medicine, Maebashi, Japan: Norimichi Koitabashi, Hideki Ishii (Gunma University Hospital Ethics Committee; the approval number HS2021-138)
- Department of Cardiology, Kokura Memorial Hospital, Kokura, Japan: Seiichi Hiramori, Kenji Ando (Kokura Memorial Hospital Ethics Committee; the approval number 21082501)
- Department of Cardiology, National Hospital Organization Kyoto Medical Center, Kyoto, Japan: Kosuke Doi, Masaharu Akao (Kyoto Medical Center Ethics Committee; the approval number 21-034)
- Department of Cardiology, Koto Memorial Hospital, Higashiomi, Japan: Hiroshi Mabuchi (Koto Memorial Hospital Ethics Committee; the approval number 2021-05)
- Division of Cardiology, Shimada General Medical Center, Shimada, Japan: Yoshiaki Tsuyuki, Hiroto Yamamoto, Takeshi Aoyama (Shimada General Medical Center Ethics Committee; the approval number R3-11)
- Department of Cardiology, Shizuoka City Shizuoka Hospital, Shizuoka, Japan: Koichiro Murata, Eri Ishikawa, Ryuzo Nawada (Shizuoka City Shizuoka Hospital Ethics Committee; the approval number 2021-10-24)
- Department of Cardiology, Hirakata Kohsai Hospital, Hirakata, Japan: Kensuke Takabayashi, Mitsunori Miho, Shoji Kitaguchi, Takeshi Kimura (Hirakata Kohsai Hospital Ethics Committee; the approval number 2021-006)
- Department of Cardiovascular Medicine, Sugita Genpaku Memorial Obama Municipal Hospital, Obama, Japan: Hisato Nakai, Yuto Miura (Obama Municipal Hospital Ethics Committee; the approval number 2021-12-14)
- Department of Cardiovascular Medicine, Graduate School of Medical Sciences, Kumamoto University, Kumamoto, Japan: Daisuke Sueta, Kenichi Tsujita (Kumamoto University Hospital Ethics Committee; the approval number 2362)
- Department of Cardiovascular Medicine, Shiga University of Medical Science, Otsu, Japan: Wataru Shioyama, Yoshihisa Nakagawa (Shiga University Ethics Committee; the approval number R2021-094)
- Division of Cardiology, Kohka Public Hospital, Koka, Japan: Tomohiro Dohke (Kohka Public Hospital Ethics Committee; the approval number 2021-9-3)

**Supplementary Appendix 2: Definitions for patient characteristics**

Hypertension was diagnosed if peripheral blood pressure was >140/90 mmHg or if the patient was taking medication for hypertension. Diabetes mellitus was diagnosed using hemoglobin A1c (HbA1c) [National Glycohemoglobin Standardization Program (NGSP), 6.5%] as the standard or was assumed if the patient was taking medication for the treatment of diabetes. Dyslipidemia was diagnosed if total cholesterol was >240mg/dL, if high-density lipoprotein cholesterol was <40mg/dL, or if the patient was taking statins. Chronic kidney disease was diagnosed if there was persistent proteinuria or if estimated glomerular filtration rate (eGFR) was <60 mL/min/1.73 m^2^ for more than 3 months. The values of eGFR were calculated based on the equation reported by Japan Association of Chronic Kidney Disease Initiative [male: 194*Scr^−1.094^*age^−0.287^, female: 194*Scr^−1.094^*age^−0.287^*0.739]. Dialysis included hemodialysis or peritoneal dialysis. Chronic heart disease was defined as persistent heart disorders including heart failure, history of myocardial infarction and atrial fibrillation. Heart failure was diagnosed if the patient had a history of hospitalization for heart failure, if the patient had symptoms due to heart failure [New York Heart Association (NYHA) functional class ≥2], or if the left ventricular ejection fraction was <40%. Autoimmune disorder was defined as immune-mediated diseases, including inflammatory bowel disease, rheumatoid arthritis and antiphospholipid syndrome. Congenital coagulation defects included protein C deficiency, protein S deficiency, and antithrombin III deficiency.

**Supplementary Appendix 3: The independent clinical event committee**

- Yuji Nishimoto, MD: Hyogo Prefectural Amagasaki General Medical Center.
- Kosuke Doi, MD: National Hospital Organization Kyoto Medical Center.
- Kensuke Takabayashi, MD: Hirakata Kohsai Hospital.
- Ryusuke Nishikawa: Graduate School of Medicine, Kyoto University.
- Kazuhisa Kaneda: Graduate School of Medicine, Kyoto University.
- Yugo Yamashita: Graduate School of Medicine, Kyoto University.
